# Immunometabolism and PI(3)K Signaling As a Link between IL-2, Foxp3 Expression, and Suppressor Function in Regulatory T Cells

**DOI:** 10.3389/fimmu.2018.00069

**Published:** 2018-01-29

**Authors:** Martin Y. Fan, Laurence A. Turka

**Affiliations:** ^1^Center for Transplantation Sciences, Department of Surgery, Massachusetts General Hospital, Boston, MA, United States; ^2^Program in Immunology, Division of Medical Sciences, Harvard Medical School, Boston, MA, United States

**Keywords:** regulatory T cells, CD25, IL-2, metabolism, aerobic glycolysis, fatty acid oxidation, PI(3)K

## Abstract

CD4^+^ Foxp3^+^ regulatory T cells (Tregs) are an essential component of immune homeostasis. Modulation of Treg function has been proposed as a means of treating autoimmune conditions and preventing rejection of organ transplants, although achieving this goal will require a detailed understanding of Treg signaling pathways. Signaling within Tregs is known to differ considerably from that observed in other T cell subsets. Of note, Tregs are the only cell type known to constitutively express CD25, the main ligand-binding subunit of the IL-2 receptor. The PI(3)K/Akt/mTOR cascade constitutes a major signaling pathway downstream of IL-2 and is closely tied to cellular metabolism. Due to increasing recognition of the links between cellular fuel usage and immune cell function, the interplay between IL-2 signaling and Treg metabolism represents an important space for exploration and a potential approach for immunomodulation. Here, we discuss how IL-2 may affect Treg metabolism *via* PI(3)K signaling, as well as the effects of altered metabolism on Treg lineage stability and suppressor function.

## Introduction

Regulatory T cells (Tregs) play a key role in maintaining immune homeostasis and in preventing the onset of autoimmune diseases (1). Modulation of Treg suppressor function is being actively explored as a promising new approach to treat autoimmunity (2–4), promote transplant tolerance (5, 6), and enhance anti-tumor responses (7, 8). Although several subsets of Tregs have been described, the best characterized is defined by the expression of CD4, CD25, and the transcription factor Foxp3 (9). The majority of circulating Tregs originate from the thymus and are termed “tTregs.” Naïve CD4^+^ T cells may also be induced to express Foxp3 in the periphery, thereby constituting a minority “pTreg” population (10) which is required for fetal tolerance (11). Although reports do not always specify which of the two populations is examined, any findings concerning “Tregs” most likely apply primarily to tTregs since they constitute the majority of Tregs in blood and secondary lymphoid organs. The importance of Tregs in maintaining peripheral tolerance is illustrated by the fact that mice (12) or humans (13) lacking Foxp3 suffer severe systemic autoimmunity. Similar, albeit less severe, autoimmune phenotypes are observed in mice (14) or humans (15) lacking CD25. Most Tregs constitutively express CD25 in addition to Foxp3, and it is generally believed that Tregs require continuous IL-2 signals through CD25 for their survival, lineage maintenance, and suppressor function (16, 17).

It is now appreciated that cell-intrinsic metabolic pathways directly impact cellular fate and function (18). Broadly speaking, aerobic glycolysis tends to support the function of pro-inflammatory cells, while fatty acid oxidation (FAO) tends to be used by anti-inflammatory cells such as Tregs (19). However, increasing evidence shows that Tregs also utilize aerobic glycolysis to achieve full suppressor function (20, 21). These metabolic programs are controlled in large part by the PI(3)K/Akt/mTOR signaling axis (22), offering multiple pharmacologic avenues to differentially target immune subsets depending on their metabolic preferences. Given the importance of CD25 for initiating PI(3)K signaling (23), in this review, we will focus on how IL-2 may interact with metabolism and the mechanisms through which metabolism influences Treg function. We touch on the difficulty of directly evaluating the interplay between IL-2 signaling, metabolism, and Treg function using existing germline knockout models and propose a means by which this issue can be addressed.

## IL-2 and Tregs

### IL-2 Signaling

**Figure 1:**
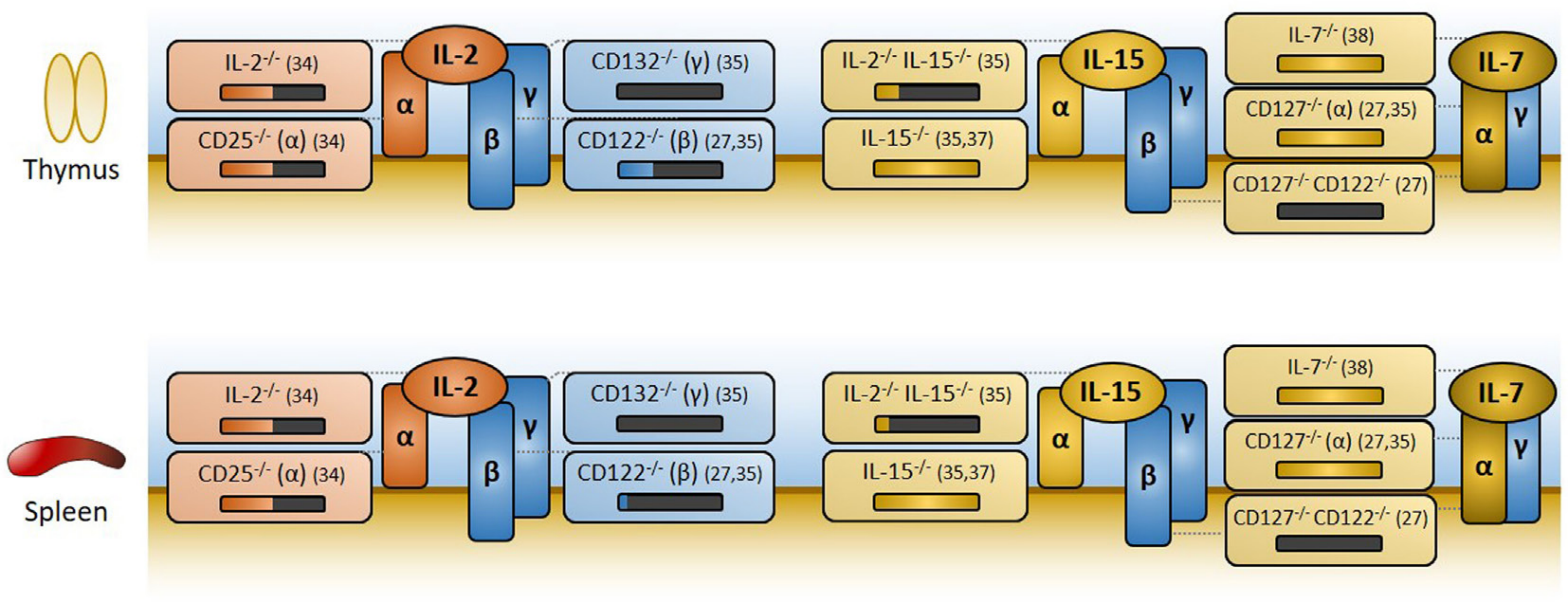
Overview of IL-2, IL-15, and IL-7 receptor components, and effects of knockouts on regulatory T cell (Treg) generation. The IL-2 and IL-15 receptors are trimers with common β and γ_c_ subunits (CD122 and CD132, respectively) that mediate signaling. High ligand affinity is conferred by their α subunit (CD25 for IL-2, CD215 for IL-15) which does not signal. The IL-7 receptor is a dimer of CD127 (α) and CD132 (γ_c_). Disruption of IL-2 signaling is detrimental to Treg development and subsequent Treg representation in the periphery, as measured by the percentage of Foxp3^+^ cells among CD4^+^ cells in the thymus and spleen, respectively. In the above figure, losses in Tregs are represented visually as black bars below knockout mouse genotypes, with relevant references for each knockout provided immediately to the right. Deletion of IL-2 or CD25 (IL-2Rα) leads to an approximate 50% reduction in Foxp3^+^ cells. In the absence of IL-2 signaling, IL-15 or IL-7 appears to compensate, albeit imperfectly. Concomitant knockout of IL-2 and IL-15, or knockout of CD122 (the shared β subunit of both IL-2 and IL-15 receptors), exacerbates defects in Treg production. Removal of signaling through all three cytokines, whether through deletion of the common gamma chain CD132 (γ_c_) or through the more targeted CD122/CD127 double knockout, virtually eliminates Treg development. Mice deficient in IL-15, IL-7, or CD127 (IL-7Rα) alone experience lymphopenia, but have normal percentages of Foxp3^+^ cells among CD4^+^ T cells and do not develop autoimmunity. Thus, IL-15 and IL-7 may partially compensate for Treg development in the absence of IL-2 signaling, but neither are required for Treg development when IL-2 signaling is fully functional.

The IL-2 receptor is composed of three subunits: CD25, CD122, and CD132, which are, respectively, referred to as the α, β, and γ_c_ (also termed the common gamma chain) subunits (24). CD122 and CD132 are the sole mediators of downstream signaling and may form a heterodimer capable of low-affinity binding to IL-2 (16) (Figure 1). The alpha subunit CD25 does not signal, but is needed for high-affinity binding to IL-2. Most Tregs constitutively express all three subunits, while conventional CD4^+^ and CD8^+^ T cells constitutively express the CD122/CD132 dimer and only express CD25 upon activation. Conventional T cells begin producing IL-2 1 h after activation (25) and constitute the primary source of IL-2 *in vivo*. IL-2 activates three major signaling axes: the STAT5, PI(3)K, and MAPK/ERK pathways. STAT5 is particularly important for Treg development, as it is necessary to initiate Foxp3 expression (26).

The receptors for two other cytokines, IL-15 and IL-7, share subunits with the IL-2 receptor and partially compensate for losses of IL-2 or CD25 (27). The IL-15 receptor is a trimer that is strikingly similar to the IL-2 receptor, sharing the CD122 and CD132 subunits used for downstream signaling. Its alpha subunit CD215, like CD25, does not signal but instead confers high ligand affinity (28). On the other hand, the IL-7 receptor is a dimer composed of CD132 and a unique alpha subunit, CD127, which is capable of activating STAT5 (29).

### IL-2 Is (Partially) Needed for Treg Development

Germline knockouts of IL-2 or its receptor components yield similar autoimmune phenotypes due to Treg deficiency (14, 30). Mice develop hemolytic anemia and colitis accompanied by thymic involution, lymph node hyperplasia, and splenomegaly, with elevated numbers of activated effector CD4^+^ and CD8^+^ T cells. Analogous findings have been reported in three clinical cases of CD25 loss (15, 31, 32), indicating that human Tregs are similarly dependent on CD25 and IL-2 signaling. These phenotypes are less severe than the scurfy phenotype resulting from Foxp3 deletion (33), most likely because the loss of IL-2 signaling also impacts effector T cells.

The fact that IL-2 and CD25 knockout mice maintain appreciable numbers of Foxp3^+^ cells in both thymus and spleen (34) indicates that IL-2 is not absolutely required for Treg development or subsequent survival, though it may be needed to achieve full suppressor function. The primary compensatory factor appears to be IL-15, as mice lacking both IL-2 and IL-15 are severely deficient in Foxp3^+^ cells (as are mice lacking either of the shared CD122 or CD132 subunits) (35, 36). In the presence of IL-2, however, IL-15 and IL-7 are dispensable for Treg development and function: IL-15^−/−^(37), IL-7^−/−^ (38), and CD127^−/−^ (27, 35) mice have normal percentages of Foxp3^+^ cells and do not develop autoimmunity.

### Post-Developmental Roles of IL-2 in Tregs

Although IL-2 signaling is an important component of Treg development (39), its roles following development are less thoroughly explored. It is generally believed that Tregs require constitutive IL-2 signals to survive and maintain Foxp3 expression, much in the same way these signals are needed during thymic development (40, 41). The role of IL-2 in Treg suppressor function has been difficult to address due to its roles in Treg survival during development. To date, the most prominent attempt to evaluate Treg function has been a Bim^−/−^ IL-2^−/−^ double knockout (42), in which targeting of the pro-apoptotic protein Bim was intended to decouple the roles of CD25 in Treg survival versus suppressor function. Although this study suggested that IL-2 is needed for full suppressor function, it should be noted that all germline knockout models of IL-2 signaling components are subject to a critical confounding factor: knockout mice develop lethal autoimmunity, which by its very nature is accompanied by immune activation and widespread inflammation. For this reason, it has been difficult to study how constitutive IL-2 signaling influences Treg lineage stability and function post development, much less study its effects on Treg metabolism.

Blocking antibody approaches can be dosed to avoid inducing autoimmunity. Although they are insufficient to address the issue of Treg function, due to off-target effects on effector T cells, these studies do not support IL-2 as a survival factor for Tregs. Anti-CD25 clone 7D4, widely used in commercial Treg magnetic isolation kits (43), induces loss of CD25 for up to 2 weeks following injection, yet Tregs persist and mice fail to develop autoimmunity (44, 45). It is critical to note that this antibody is distinct from anti-CD25 clone PC61, commonly as a tool to deplete Tregs *in vivo*, which is now recognized to act *via* opsonization for phagocytosis rather than through IL-2 deprivation (46–48).

## PI(3)K Signaling in Tregs

Because the role of IL-2-induced STAT5 signaling in Treg development has been reviewed extensively (16), here we focus on how lineage stability and suppressor function are influenced by metabolism in mature, post-developmental Tregs. PI(3)K catalyzes the conversion of PIP2 (PtdIns-4,5-P2) to PIP3 (PtdIns-3,4,5-P3) to permit activation of kinases with plextrin homology domains, most notably Akt. Targets of Akt include the protein translation regulator complex mTOR, which promotes cellular growth and survival (49). Thus, one major downstream effect of PI(3)K signaling is induction of aerobic glycolysis, which is increasingly emerging as a key control mechanism of Treg function (see below). The lipid phosphatase PTEN, which dephosphorylates PIP3 back into PIP2, and the protein phosphatase PHLPP, which dephosphorylates Akt, are the primary negative regulators of PI(3)K activity in T cells (50, 51). Excessive PI(3)K activity is detrimental to Tregs since loss of PTEN in mice (52, 53), loss of PHLPP in mice or in human cell culture (51), and induced Akt activation in human cell culture (54) all lead to Treg lineage instability and loss of suppressor function. Tregs may receive signals from three sources which would normally induce strong PI(3)K signaling: the TCR, CD28, and the IL-2 receptor (23). To prevent excessive PI(3)K signaling from these sources, Tregs express high levels of PTEN (55, 56) and PHLPP (51).

## Treg Metabolism

### Glycolysis

Following immune cell activation by antigen or inflammatory signals, aerobic glycolysis and fatty acid synthesis are rapidly induced to support cell proliferation and cytokine secretion (57). This is reflected in the metabolic profiles of relevant immune subsets: effector T cells such as Th1, Th2, and Th17 cells show increased glycolytic rates following activation, as do effector CD8^+^ T cells. Tregs, like memory CD8^+^ T cells, rely on FAO for their basal metabolism but utilize some degree of aerobic glycolysis to properly execute their suppressor functions.

**Figure 2:**
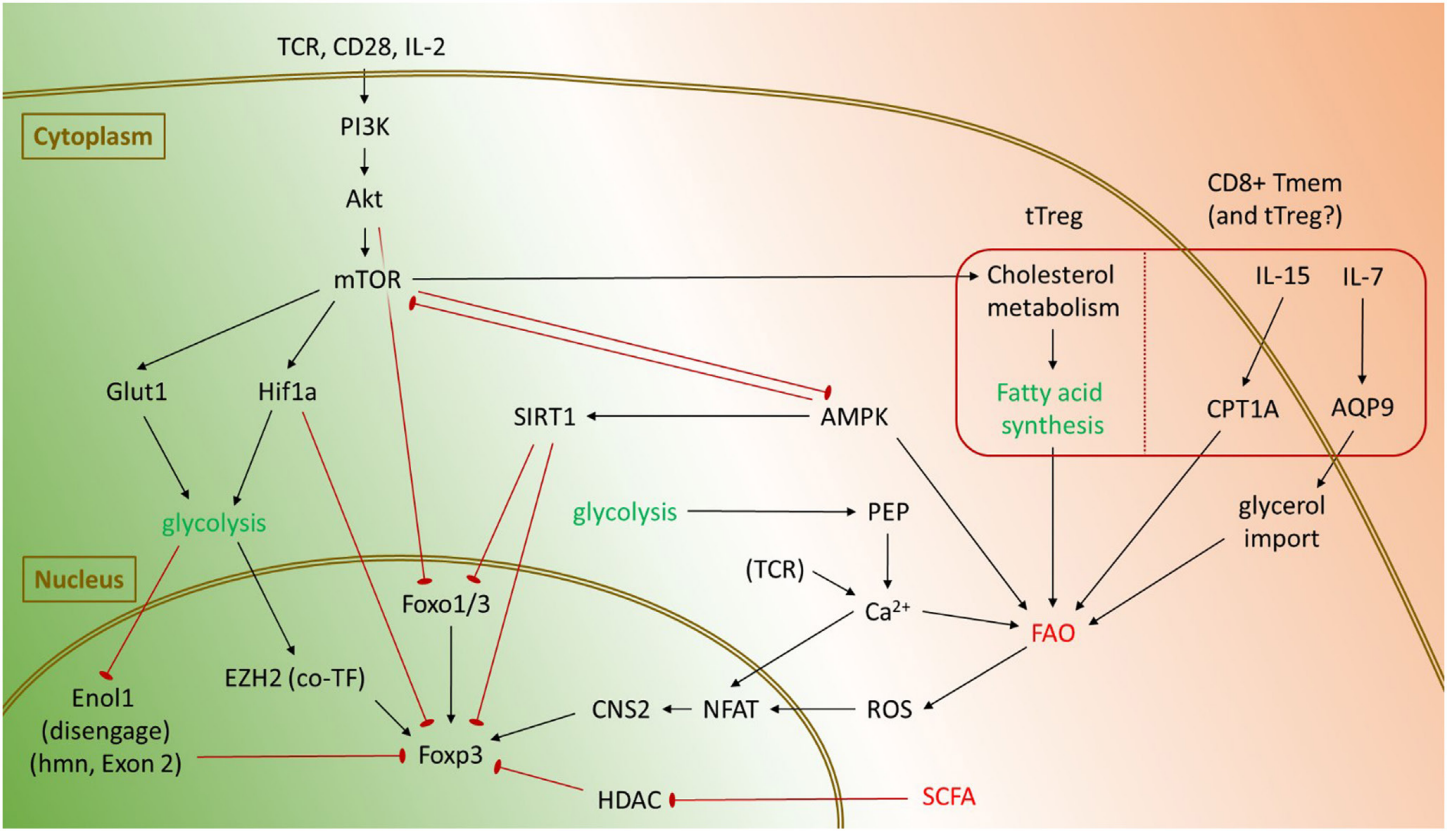
Pathways promoting glycolysis and fatty acid oxidation (FAO) in regulatory T cells (Tregs), and known mechanisms affecting Foxp3. Glycolysis is primarily activated in Tregs through mTOR and tends to suppress Foxp3 expression and Treg lineage stability. Activation of the PI(3)K/Akt/mTOR signaling axis inhibits Foxo transcription factors and promotes activation of Hif-1α, which can directly target Foxp3 for degradation. However, under certain conditions, glycolysis also promotes Foxp3 expression. By disengaging Enolase 1 from its nuclear role, glycolysis enables expression of the Foxp3-E2 splice isoform in humans. Glycolysis also represses microRNAs such as miR-101 and miR-26a to enable expression of EZH2, which is a cotranscription factor for Foxp3. Tregs generally rely upon FAO for their metabolic needs. In the gut, short-chain fatty acids (SCFA) inhibit histone deacetylases (HDACs) to promote Foxp3 expression and conversion of naïve CD4^+^ T cells into pTregs. Under certain conditions, FAO may also impinge upon Treg lineage stability. Sirt1 may repress Foxp3, either through direct deacetylation of Foxp3 or by targeting Foxo transcription factors. In CD8^+^ memory T cells, cytokines such as IL-7 and IL-15 promote uptake of fatty acid precursors and increased FAO, respectively. It remains to be seen whether similar processes occur in Tregs as well. Both glycolysis and FAO can also promote Foxp3 expression through an NFAT-dependent mechanism.

Beyond mere association with immune activation, several causal links have emerged between inflammatory stimuli, glycolysis, and Tregs (Figure 2). In T cells, signals through the TCR, CD28, or IL-2 activate the PI(3)K/Akt/mTOR cascade (58), which induces expression of the glucose transporter Glut1 to facilitate increased glycolysis (59). Akt also inhibits Foxo1 and Foxo3 transcription factors which are important for Foxp3 gene expression (60–62). mTOR engages Hif-1α, which may also be independently activated through toll-like receptor signaling, to promote the expression of key glycolytic genes (63). Hif-1α may also directly bind Foxp3 and target it for proteasomal degradation (64). Reciprocally, forced Foxp3 expression is sufficient to suppress glycolysis and promote FAO *in vitro* (20). Treg effector molecules such as CTLA4 and PD-1 suppress glycolysis in CD4^+^ T cells by activating PTEN to antagonize PI(3)K signaling and subsequent glycolysis, with PD-1 also actively promoting FAO by increasing expression of CPT1A (65). These data suggest that elevated glycolysis is detrimental to Treg lineage stability and suppressor function.

However, most studies showing detrimental effects of glycolysis on Tregs were performed *in vitro*, where T cell activation and glycolysis were driven to their maximum extent. Under certain conditions, glycolysis actually supports Foxp3 expression, promotes Treg proliferation, and potentiates suppressor function. Among *in vitro* induced human Tregs, the glycolytic enzyme Enolase-1 binds the Foxp3 promoter and its CNS2 regions. This represses transcription of a splice isoform containing Exon 2 (Foxp3-E2), which is needed for optimal Treg suppressor function. Engaging glycolysis forces Enolase-1 into the cytoplasm, thereby allowing transcription of Foxp3-E2 (66). Glycolysis also favors expression of the histone methyltransferase EZH2 by repressing inhibitory microRNAs such as miR-101 and miR-26a (67). EZH2 in turn binds Foxp3 to assist suppression of target genes (68), although no experiment has yet confirmed glycolysis-dependent EZH2 expression is essential for Treg lineage stability. The glycolytic metabolite phosphoenol pyruvate (PEP) can also increase Foxp3 expression through an NFAT-dependent mechanism. By inhibiting the calcium ATPase SERCA, PEP increases intracellular Ca^2+^ levels to promote nuclear translocation of NFAT, which facilitates interactions between the Foxp3 promoter and its CNS2 regions (69, 70).

A recent study suggests a possible resolution of these conflicting roles for glycolysis in Tregs. Using a Glut1 transgene to increase glucose uptake and glycolysis, the authors found that although elevated glycolysis boosts tTreg proliferation, it comes at the cost of their ability to execute suppressor functions (20). This suggests that for optimal Treg activity, a balance must be struck between the cell activating effects of glycolysis with its negative effects on the lineage.

### Fatty Acid Oxidation

Fatty acid oxidation is generally associated with an anti-inflammatory phenotype and maintenance of Treg lineage stability. One mechanism is through simple antagonism of glycolysis: Tregs express high levels of AMPK, which simultaneously promotes FAO while inhibiting mTOR and subsequent glycolysis (71). In the gut, short-chain fatty acids are also known to inhibit mTOR (72). They have the added benefit of stabilizing pTregs by inhibiting histone deacetylases (HDACs) such as HDAC6 and HDAC9 which would otherwise inhibit Foxp3 expression (73, 74). Reactive oxygen species generated as a byproduct of oxidative phosphorylation have been shown to promote Foxp3 stability by increasing activity of the transcription factor NFAT, which binds the CNS2 enhancer of Foxp3 (70, 75). In addition, Foxp3 may experience post-transcriptional modifications such as acetylation, which prevents Foxp3 from being targeted for degradation thereby increasing its half-life (76). Foxp3 acetylation is dependent on nuclear availability of acetyl-CoA, whose supply is increased upon breakdown of fatty acids. As with glycolysis however, under certain conditions FAO may antagonize Treg lineage stability. FAO promotes an increased NAD^+^/NADH ratio, which elevates the activity of the deacetylase SIRT1 (77). By deacetylating Foxp3, SIRT1 promotes Foxp3 poly-ubiquitination and subsequent proteasomal degradation (78).

pTregs and tTregs diverge considerably in their execution of FAO: although pTregs generally rely upon exogenous fatty acids for their metabolic needs (79), it is uncertain whether tTregs can import exogenous fatty acids *in vivo* (18). While the coming years will likely clarify this issue, available literature suggests one peculiar metabolic feature among tTregs. One of the major roles for mTOR signaling in tTregs is to drive synthesis of endogenous fatty acid stores, primarily along cholesterol biosynthetic pathways (80). Whether these endogenously synthesized fatty acids are then used for energy is not known, although these specific pathways are needed for tTreg proliferation and optimal suppressor function. Memory CD8^+^ T cells constitute the only major T cell subset known to synthesize endogenous fatty acids for subsequent FAO *in vivo* (18, 81) and rely in part on IL-7 and IL-15 to regulate these processes. IL-7 induces expression of the channel protein aquaporin 9, which facilitates glycerol import for fatty acid synthesis (82). IL-15 increases FAO by stimulating mitochondrial biogenesis and elevating expression of CPT1a, a key regulator of FAO (83). Given that Tregs appear to rely on IL-7 and/or IL-15 in the absence of IL-2, we speculate that tTregs from IL-2 or CD25 knockout mice may experience a shift from glycolysis to FAO, possibly with an associated loss of suppressor function. Whether similar events might occur in pTregs is unknown, although prior literature (11) suggests a loss of suppressor function in pTregs would result in increased fetal resorption among any IL-2 or CD25 knockout mothers which reach breeding age.

### Therapeutic Interventions

One of the most exciting prospects of immunometabolism is developing therapeutic interventions which can selectively target T cell subsets. Since activated effector T cells are more reliant on glycolysis than Tregs, studies have examined whether inhibiting glycolysis might improve outcomes in mouse models of autoimmunity and transplant rejection. Blocking glycolysis with 2-DG (a competitive inhibitor of hexokinase), or with dichloroacetate (an inhibitor of PDHK isoforms) reduced the severity of experimental autoimmune encephalomyelitis with associated decreases in the percentage of Th17, but not Treg, cells (63, 84). Similar outcomes were reported following inhibition of another glycolytic enzyme, acetyl-CoA carboxylase 1 (ACC1), with soraphen A or with T cell specific genetic deletion of ACC1 (79). Furthermore, treatment with metformin (an agonist of AMPK, which increases fatty acid uptake and oxidation) reduced airway inflammation and fibrosis in a murine asthma model (85). In the transplant setting, treatment with 2-DG, metformin, and a glutamine uptake inhibitor DON prolonged allograft survival in heart and skin transplants, in part by suppressing the proliferation of antigen-specific T cells and by increasing the relative frequency of Tregs (86).

Conversely, interventions that promote glycolysis enhance immune function, presumably by increasing the proliferation and function of effector T cells while inhibiting Treg function. Pharmacological blockade or genetic loss of PTEN leads to Akt-dependent inhibition of Foxo3a and subsequent loss of Foxp3 and tumor regression (87). Furthermore, increasing glycolysis through forced expression of the glucose transporter Glut1 in Tregs exacerbated pathology in an adoptive transfer model of colitis (20). Tregs recovered from this system were also found to have lower levels of Foxp3 protein.

## Conclusion and Future Directions

The metabolic state of Tregs defies simple categorical explanations with regard to glycolysis and FAO. Although elevated glycolysis is generally associated with immune activation and can be detrimental to Treg lineage stability and function, controlled levels of glycolysis are necessary to sustain the same processes. The list of known links between metabolism and Treg function is far from complete, and the coming years will likely reveal other metabolic enzymes with moonlighting roles in Treg biology. In particular, the “futile cycle” approach of tTregs to FAO, and its preference for cholesterol synthesis may be a promising area of discovery.

Metabolic interventions offer a promising new approach to modulating Treg function and may be used to fine-tune therapies targeting other signaling pathways or used as a primary therapy in their own right. Of note, while there is clear potential for interplay between IL-2 signaling and immunometabolism through the PI(3)K/Akt/mTOR signaling cascade, to date no studies have specifically evaluated the effects of IL-2 signaling on Treg metabolism. In part, this is due to the inadequacies of germline knockout models to address this question. As mentioned before, such knockouts experience an autoimmune environment in which immune cells are already highly active and presumably glycolytic. It would be more appropriate to use a model in which Tregs can be inducibly made to lose IL-2 signaling while maintaining immune homeostasis. A tamoxifen-inducible CD25 knockout, with tamoxifen dosage adjusted to leave enough CD25-competent cells to prevent autoimmunity, would be well suited for this approach. Such studies would lay the framework for combination treatments in which metabolic interventions would be used with existing therapies such as CD25 blockade.

## Author Contributions

MF and LT conceived of and wrote the review.

## Conflict of Interest Statement

LT discloses personal or family financial interests with Third Rock Ventures, Tango Therapeutics, and Neon Therapeutics. LT has served as a consultant to Lycera, Solid Bio, UCB, and Intellia. M.F declares that the research was conducted in the absence of any commercial or financial relationships that could be construed as a potential conflict of interest.
